# Assessing the Dynamics of COVID-19 Morbidity and Mortality in Response to Mass Vaccination: A Comparative Study Between Saudi Arabia and the United Kingdom

**DOI:** 10.7759/cureus.33042

**Published:** 2022-12-28

**Authors:** Elham M Khatrawi, Anwar A Sayed

**Affiliations:** 1 Department of Medical Microbiology and Immunology, Taibah University, Madinah, SAU; 2 Department of Surgery and Cancer, Imperial College London, London, GBR

**Keywords:** united kingdom, vaccination, saudi arabia, public health, herd immunity, covid-19

## Abstract

Background

Since the beginning of the coronavirus disease 2019 (COVID-19) pandemic, efforts have been in place to tackle the infection. Mass vaccination programs were regarded as the sole solution to end the pandemic. Here, we compare the differential impact of mass vaccination programs in Saudi Arabia (SA) and the United Kingdom (UK) on COVID-19 morbidity and mortality to determine whether vaccines were solely responsible for the changes observed in the disease dynamics.

Methodology

We retrospectively collected the number of new cases and deaths throughout 2021 in both SA and the UK. Similarly, the number of vaccine doses delivered in both countries was collected and compared.

Results

Through 2021, the percentage of daily COVID-19 cases was significantly less in SA than in the UK; however, the percentage of deaths was higher in SA. Interestingly, the percentage of daily cases was significantly reduced in SA upon vaccination. The vaccination coverage of both the first and second doses was higher in the UK compared to SA, and, consequently, the percentage of deaths was significantly reduced in the UK compared to SA.

Conclusions

The UK vaccination program succeeded in curbing the number of daily deaths compared to SA. SA had better control over the percentage of daily cases, primarily due to the restrictive measures and vaccination, such as the imposed social distancing and mandatory face masks.

## Introduction

The novel coronavirus disease 2019 (COVID-19) hit the world severely, and the World Health Organization (WHO) declared it a pandemic in March 2020, a status that has remained until this day [1]. Countries were racing to limit COVID-19 spread and contain its detrimental sequelae. For example, Saudi Arabia (SA) introduced several strict progressive measures, such as a complete travel ban and 24-hour curfews [2]. Despite the different measures taken across countries, all hopes of surviving and overcoming the pandemic were hinged on developing an effective vaccine that develops a protective immunity that prevents the infection and its transmission between people.

Pfizer/BioNTech and AstraZeneca vaccines were the earliest vaccines developed against COVID-19. These two vaccines demonstrated considerable efficacy in animal studies and clinical trials, indicated by the generated anti-COVID-19 antibodies and the development of COVID-19-specific T cells. However, their real-world efficacy was still to be determined. As soon as these two vaccines became available, SA obtained them and started a mass vaccination program to tackle the infection [3]. Early reports demonstrated that a single dose of the vaccine was insufficient to prevent the infection nor spread to others [4]. Concurrently, social measures were started to be lifted, and tight restrictions were eased.

Despite the mass vaccination programs, including first and second doses of the vaccines, third and fourth waves of COVID-19 cases continued in different countries, including SA and the United Kingdom (UK) [5,6]. Such an observation raises the question of whether the vaccines have a similar impact on the disease dynamics and whether the current vaccines are of medical significance to public health in SA and the UK.

In this study, we analyzed the publicly available data on the number of severe cases, reflected by the number of deaths and the number of vaccines (first or second) in SA and the UK, and how they impact each other. Then, we compared these parameters between SA and the UK to determine whether vaccines were solely responsible for the changes observed in the disease dynamics.

## Materials and methods

The study is based on COVID-19 data that is publicly available. Information on the number of new cases recorded and daily deaths in SA were collected from the Saudi Arabian Ministry of Health website [5]. Similar data regarding the UK population were obtained from the WorldoMeter Website [6]. All vaccination information, namely, the vaccination coverage of both the SA and the UK populations, was obtained from WorldoMeter. These obtained data were compared with the official and governmental authorities’ websites in both these countries and they were found to be similar.

The data collected covered 33 time points from the beginning of January 2021 until the end of November 2021. The comparison of the daily new cases was based on the percentage of daily new cases from the total number of populations to ensure the consistency of the comparison between the SA and UK populations, which are almost 2.5 times more than the SA population. The percentage of the daily new cases was calculated by dividing the total number of cases on a particular day by the total number of populations.

To compare the COVID-19 mortality between the two countries, the percentage of deaths, rather than absolute numbers, was calculated and compared. The percentage of deaths was obtained by dividing the daily deaths by the number of COVID-19-infected individuals. The total populations of both SA and the UK were obtained from the WorldoMeter website.

We selected these two countries specifically as we wanted to assess the dynamics of COVID-19 morbidity and mortality in response to mass vaccination in both developed and developing countries. Furthermore, SA has been more restrictive in dealing with the pandemic compared to the UK, and these two counties are considered high-income countries.

Statistical analysis

The data were analyzed using GraphPad Prism 5 software (GraphPad Software, Inc, La Jolla, USA). The two-way analysis of variance (ANOVA) followed by the Bonferroni post-test to compare the mean differences between groups that were split into two independent variables were exploited to identify statistical significance. Furthermore, Student’s t-test was used to compare the two groups. A Spearman correlation test was used to find the association degrees of two variables. A p-value of <0.05 denoted statistical significance. Due to the nature of the study, the institutional review board’s ethical approval was waived by the Taibah University College of Medicine Research Ethics Committee.

## Results

The Dynamics of COVID-19 infection in response to vaccination in SA and the UK

**Figure 1:**
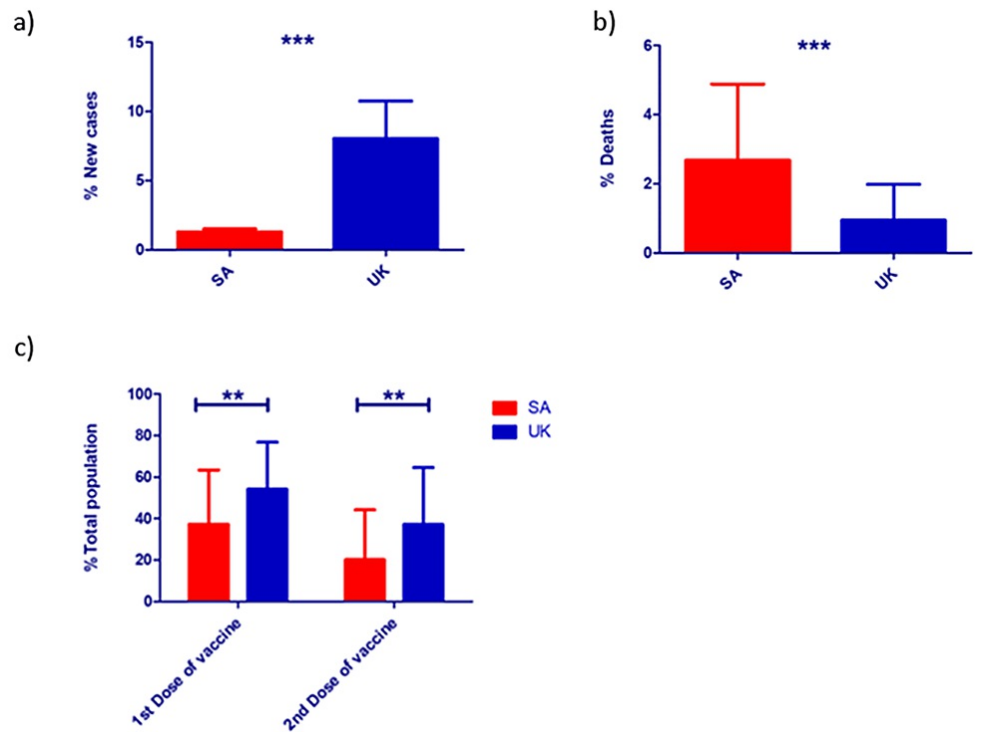
Comparisons of different COVID-19 infection aspects between SA and the UK. The differences in (a) the percentage of new cases, (b) the percentage of deaths between SA (red) and the UK (blue), and (c) the overall percentage of Saudi versus British populations taking the first and second doses of COVID-19 vaccines. Student’s t-test was used to perform the analysis (**p < 0.01, ***p < 0.001). Data represent the mean ± SD. COVID-19 = coronavirus disease 2019; SD = standard deviation; SA = Saudi Arabia; UK = United Kingdom

The percentages of COVID-19 daily new cases were analyzed in both countries. The UK had a significantly higher percentage of daily new cases in comparison to SA (8% vs. 1.3%: p < 0.001) (Figure 1a). In addition, the percentage of deaths resulting from infection by COVID-19 was significantly higher in SA compared to the UK (2.7% vs 0.9%: p < 0.001) (Figure 1b). The percentage of the total population that received the first and second doses of COVID-19 immunization was demonstrated in both countries, and there were significant differences in the percentage between the SA and the UK populations. A higher percentage of the UK population got vaccinated with the first (54.2%) and the second (37.2%) doses. In contrast to the British population, only 37.4% and 20.2% of the Saudi Arabian population were immunized with the first and second doses of COVID-19 vaccines, respectively (Figure 1c).

**Figure 2:**
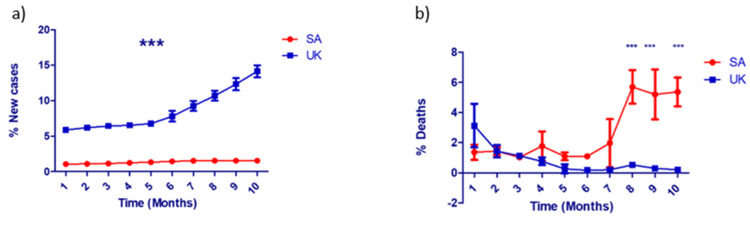
The COVID-19 morbidity and mortality dynamics in relation to the vaccination status. The percentage of (a) new cases over months after the administration of the COVID-19 vaccine in SA (red) versus the UK (blue), and (b) deaths over months after the administration of the COVID-19 vaccine in both SA and the UK. Each month is represented by three time points. Data represent mean ± SD. Two-way ANOVA test followed by Bonferroni multiple-comparison tests were used (***p < 0.001). COVID-19 = coronavirus disease 2019; SD = standard deviation; ANOVA = analysis of variance; SA = Saudi Arabia; UK = United Kingdom

We compared the percentage of new COVID-19 cases and deaths resulting from this infection between SA and the UK across the months after the beginning of COVID-19 immunization. The percentage of COVID-19 cases in the UK increased in the months after the population was vaccinated, especially after the fifth month (Figure 2a). In the UK, the percentage of new cases almost remained constant, and it has been demonstrated that there were significant differences in the increase in the percentage of new cases in SA in contrast to the UK across months of COVID-19 vaccine administration. Moreover, we compared the percentage of deaths resulting from COVID-19 infection between SA and the UK in months following the complete vaccination. The percentage of deaths increased in SA after six months of getting the vaccine and increased slightly in the UK after seven months of the vaccine, and returned back to decrease after nine months. In SA, the percentage of deaths was significantly higher compared to the UK in the eight, nine, and ten months after the vaccine administration (Figure 2b).

The effect of COVID-19 vaccines on the daily number of cases as well as on the daily number of deaths in SA and the UK

**Figure 3:**
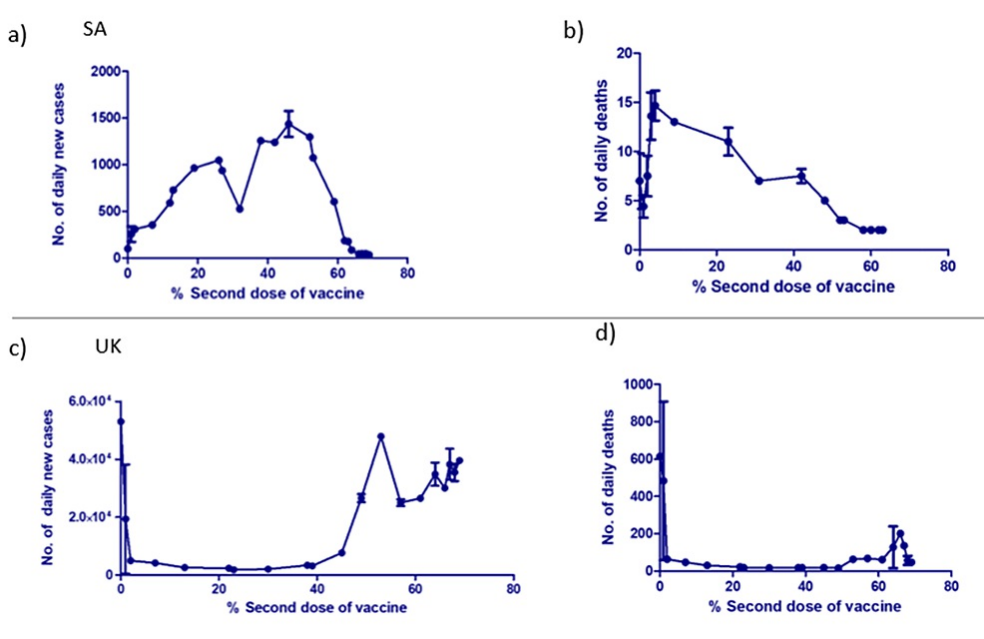
Daily number of new cases and daily number of new deaths in response to the administration of the second dose of the COVID-19 vaccine in SA versus the UK. (a) The number of daily new cases, (b) the number of daily deaths in SA, (c) the number of daily new cases in the UK, and (d) the number of new daily deaths in the UK in response to the administration of the second dose of the COVID-19 vaccines. Student’s t-test was used to perform the analysis (**p < 0.01, ***p < 0.001). Data represent the mean ± SD. COVID-19 = coronavirus disease 2019; SD = standard deviation; SA = Saudi Arabia; UK = United Kingdom

To assess the effectiveness of the COVID-19 vaccines and their impact on the number of cases and deaths, we plotted the number of daily new cases and the number of daily deaths in relation to the percentage of those who received the two doses of the vaccine. Such a comparison was possible as both SA and the UK population received COVID-19 vaccination made of two doses, e.g., Pfizer-BioNTech and AstraZeneca vaccines. The number of daily new cases dramatically decreased when 53% of the Saudi population got vaccinated with the second dose of the vaccine (Figure 3a). Moreover, the number of daily deaths started to decline when 48% of the Saudi population received the second dose of immunization (Figure 3b). On the other hand, the number of new COVID-19 cases and daily deaths in the British population surprisingly decreased rapidly after only 2% of the population was immunized with the second dose (Figures 3c, 3d). However, it was observed that the curve of daily COVID-19 cases increased significantly after 45% of the population was vaccinated. Further, the number of daily deaths increased when 61% of the population received the second dose but dropped rapidly when 67% were immunized with the second dose.

The correlation between the administration of the second dose of COVID-19 immunization and the number of daily new cases and deaths

**Figure 4:**
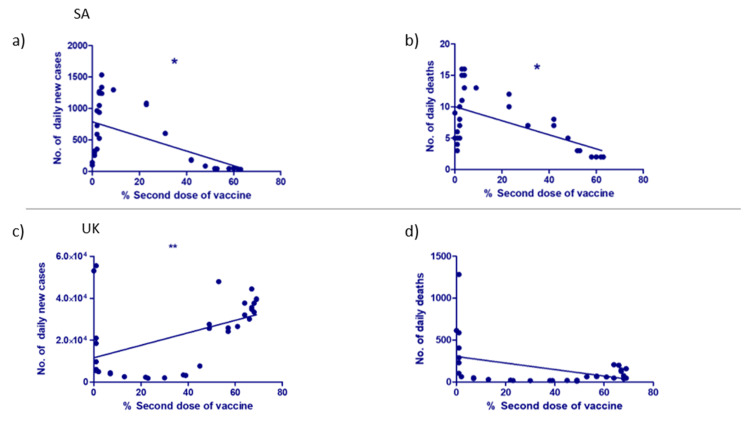
The association between the administration of the second dose of the COVID-19 vaccine and the daily cases and deaths. (a) The number of daily new cases in SA, (b) the number of daily deaths in SA, (c) the number of daily new cases in the UK, and (d) the number of daily deaths in the UK. Spearman correlation test has been exploited (*p < 0.05, **p < 0.01). COVID-19 = coronavirus disease 2019; SD = standard deviation; SA = Saudi Arabia; UK = United Kingdom

The association between the percentage of the total population who received the second dose of COVID-19 immunization and the number of daily new cases of COVID-19 infection was compared in both countries. In SA, there was a significant indirect correlation between the number of daily cases and the number of daily deaths, with the percentage of the total Saudi population receiving the second dose of vaccine (Figures 4a, 4b). On the other hand, the trend was different in the UK when compared to SA. In the UK, the correlation between the percentage of the population who were vaccinated with the second dose and the number of daily COVID-19 new cases was directly correlated significantly (Figure 4c). This could be attributed to the lack of strict adherence to the precautionary measures in the UK, which subsequently gave rise to the new variant of COVID-19 that spared faster than the old one. On the other hand, the number of daily deaths was inversely correlated with the percentage of the second dose of immunization (Figure 4d), similar to the trend in SA.

## Discussion

In our study, it has been illustrated that SA had a smaller percentage of overall daily new cases of COVID-19 infection compared to the UK, although SA had a high percentage of daily deaths as a result of this infection in contrast to the UK. The overall low percentage of COVID-19 infection in SA might be explained by the fact that SA applied stringent precautionary measures to tackle the spread of the infection from the beginning of the pandemic in contrast to the UK. At the commencement of the pandemic, in SA, stringent safety measures were implemented. In addition to the safety measures, traveling outside the country was completely banned for citizens for long period in SA in contrast to the UK. Travelers played an important role, especially when they returned from India with mutations and asymptotic infection [2,7]. However, the government eased these measures and tried to return cautiously and safely to everyday life.

We have also shown that the UK had a high percentage of its population immunized with the first and second doses of COVID-19 vaccination. Such vaccination coverage could be the reason behind the pandemic’s low percentage of overall deaths compared to SA. Multiple studies supporting this finding have shown that the full dose of COVID-19 vaccines can prevent severe disease and hence deaths [8-10].

It has also been noted that the percentage of COVID-19 daily cases and deaths rose in SA five and six months, respectively, after vaccine administration. It has been suggested to be due to immunity reduction after the first dose [11], as well as as a result of the emergence of a new variant called delta, which appeared initially in India in December 2020 [10]. This variant was the dominant one before it spread to other countries, reaching the UK through travelers from India [12,13]. This variant likely requires a booster dose of the vaccine to be more effective in tackling the infection and reduce the severity of the disease, and thus the deaths [14-18].

In the UK, the situation was different where the percentage of cases over months of COVID-19 vaccine administration and the percentage of daily new cases remained almost constant. Additionally, the percentage of deaths declined over months of getting the vaccine. Comparatively, there were significant differences between SA and the UK in the percentage of cases and deaths over months eight, nine, and ten after receiving the shot. Concurrent to many studies, administering the second dose of the COVID-19 vaccine can reduce daily new cases of COVID-19 and related deaths [19,20].

In our study, there was an inverse correlation between the percentage of the population receiving the second dose of immunization and the number of daily COVID-19 cases and deaths in SA. Moreover, the percentage of deaths in the UK was inversely correlated with the percentage of the population vaccinated with the second dose. However, in the UK, there was a direct correlation between the percentage of the population that got the second shot of the vaccination and the number of daily new cases.

COVID-19 vaccination was vital to tackling and reducing the impact of the pandemic, i.e., reducing COVID-19 morbidity and mortality [19,20]. However, the COVID-19 pandemic, unlike other previous ones, was associated with the spread of misinformation globally, which was facilitated through social media [21]. Such misinformation included conspiracy theories, and the pandemic was intended to be caused so pharmaceutical companies could make huge profits [22]. The use of social media and subsequently trusting them hindered people’s willingness to be vaccinated [23], as well as their adherence to the precautionary measures set by the government [2].

We showed that following the precautionary measures to stay safe, especially with the emergence of COVID-19 variants, made it difficult to stop the pandemic’s spread with only the vaccine. Although this study was carefully planned and implemented, it is not without limitations. The study covers a period between January and the end of November 2021. The study could be improved by extending the study period to include 2022. Another noted limitation is the limited collected data, i.e., three data points were collected for each month. Although more data could be collected, we resorted to three data points to ensure consistency regarding the days collected across all months between the two countries. Finally, because our data were obtained from government sources, there was no stratification of cases by age, gender, or any other demographic characteristics.

## Conclusions

The percentage of COVID-19 daily cases was significantly reduced in SA upon vaccination. The vaccination coverage of both the first and second doses was higher in the UK compared to SA, and, therefore, the number of deaths was significantly reduced in the UK compared to SA. Mass vaccination programs succeeded in both SA and the UK as the number of daily cases and deaths decreased at first. However, vaccinations may not be sufficient to curb the infection’s spread completely. The different applications of protective measures such as social distancing and mandatory face masks in public led to the differences observed in daily infections and deaths. Therefore, it is recommended to receive the required doses of COVID-19 vaccination according to your age and health status alongside applying safe precautionary measures such as mask-wearing, especially in overcrowded places, regular hand hygiene, and keeping as much distance as possible from the sick or COVID-19-positive individuals.
